# Device Access, Gender, and Academic Year Influence E-Learning Patterns in Indian Medical Education

**DOI:** 10.7759/cureus.106387

**Published:** 2026-04-03

**Authors:** Md Jamil, Hanifa Akhtar, Bhupen Barman, Ellora Barman

**Affiliations:** 1 Department of General Medicine, All India Institute of Medical Sciences, Guwahati, Guwahati, IND; 2 Department of Otorhinolaryngology, All India Institute of Medical Sciences, Guwahati, Guwahati, IND; 3 Department of Library Science, All India Institute of Medical Sciences, Guwahati, Guwahati, IND

**Keywords:** digital media use, education, graduate medical, online learning, self directed learning

## Abstract

Purpose: E-learning patterns and resource utilization for learning different tasks and domains vary. As data on e-learning from the northeast part of India are scarce, understanding learner characteristics, device access, platform preferences, and e-learning behavior of undergraduate students from the region is essential to develop context-sensitive digital educational strategies.

Methods: A cross-sectional questionnaire-based study was conducted among 176 Bachelor of Medicine and Bachelor of Surgery (MBBS) students at a tertiary care institute to investigate the pattern of device access, e-learning resources, benefits, and barriers. Associations between gender, MBBS phase, device ownership, and e-learning use were examined using chi-square tests and multivariate logistic regression to identify independent predictors.

Results: Smartphone ownership (161, 91.5%) and laptop ownership (82, 46.6%) were high. E-learning was broadly used for theory (168, 95.5%), practical skills (145, 82.4%), clinical cases (141, 80.1%), and assessments (94, 53.4%). YouTube videos were predominantly used for practical and surgical technique learning in 124 (70.5%) and 98 (55.7%), respectively. Awareness of the institutional learning management system (LMS) and National Programme on Technology Enhanced Learning (NPTEL) course attendance was low (22, 12.2%), while paid course attendance was substantial (107, 60.8%). Female students used e-learning significantly more for assessment, and senior MBBS students showed higher usage for clinical case learning and online assessments. Logistic regression revealed smartphone and laptop ownership and gender as significant predictors for engagement in assessment-related learning.

Conclusion: E-learning adoption among medical undergraduates is device-dependent, with gender and academic phase influencing domain-specific engagement. The preference for paid courses and multimedia and social media-based resources highlights the self-directed learning behavior of the students and the need for the promotion of institutional and free national platforms.

## Introduction

The digital transformation of medical education has made e-learning a core component of undergraduate training worldwide [1,2]. Online platforms, mobile applications, learning management systems, and multimedia resources are integral for knowledge, clinical skill acquisition, and self-assessment [2]. Medical students engage in e-learning in variable task-oriented ways, like selecting different resources for different domains. This shift is well described by self-directed learning and connectivism frameworks among the medical undergraduates [3].

In high-income countries, institutional learning management systems and digital resources are embedded within the curriculum, while commercial exam-focused courses are widely used for advanced test preparations [3]. Students in this context demonstrate sophisticated self-directed learning behaviors by choosing resources as per their perceived utility for a specific task or domain [4].

In low- and middle-income countries (LMICs), the adoption of e-learning is determined by both opportunity and constraints. Smartphones are widely owned, but reliable Internet access, laptops or tablets, and robust institutional digital infrastructure remain uneven [5]. Students often rely on social media and free multimedia platforms [6-8]. Within this context, gender and academic phase can introduce further variations. Female students in some settings face additional constraints, while senior students tend to use commercial exam-focused platforms even when the free national resources exist [8-10].

India reflects many of these LMICs' features alongside rapid digital expansion. National initiatives such as the National Program on Technology Enhanced Learning (NPTEL) [11] and the Indian Council of Medical Research (ICMR) short-term studentship (STS) [12] aim to offer free structured learning and early research exposure, whereas commercial platforms like Marrow and Prep Ladder provide exam-aligned content [5,7]. Prior Indian studies have shown high acceptance of e-learning and near-universal access to smartphones among medical undergraduates, patterns of choice of online resources, perceived relevance, and barriers [8]. But data on predictors of e-learning patterns from India remain underrepresented in the literature. This study therefore aims to characterize the utilization of e-learning among medical undergraduates at a tertiary care institute in Northeast India across learning domains and to examine how gender, MBBS phase, and device ownership predict adoption of e-learning. Grounded in self-directed learning and connectivism, our study aims to generate context-sensitive data from the northeastern part of India.

## Materials and methods

Study design and setting

This was a descriptive cross-sectional questionnaire-based (Appendix) study conducted among undergraduate medical students at a tertiary care teaching institute in Northeast India. The study was carried out over a period of six months between July and December 2024.

Study population and sampling

All MBBS students enrolled from the first to the final year during the study were eligible to participate. Students who were absent on the day of data collection or did not provide consent were excluded. A total of 176 students completed the survey and were included in the final analysis. Data were collected using a structured self-administered questionnaire developed by the investigators to assess access to the digital devices, patterns of e-learning across theoretical, practical, and clinical domains, awareness and utilization of specific platforms (social media, learning management system [LMS], NPTEL, paid courses, and ICMR STS), perceived benefits, barriers, and overall view towards e-learning.

To ensure the questionnaire's validity and reliability, a comprehensive validation process was undertaken. Initially, a thorough literature review was conducted to gather relevant information related to e-learning patterns among MBBS students. The questionnaire was then subjected to content validation by five experts in medical education and research methodology. These experts assessed the clarity, relevance, and completeness of the questions, providing feedback on the questionnaire's structure and content. Modifications were made based on their suggestions to improve face and content validity.

Following content validation, the questionnaire underwent pilot testing with a sample of 10 MBBS students who were excluded from the final study sample. The pilot test aimed to ensure that the questions were easily understood and that the response options were comprehensive. Feedback from the pilot test was used to refine the questionnaire further, enhancing its clarity and relevance.

The questionnaire comprised sections on demographic details; access to devices and internet; purpose of e-learning; patterns of resources and platform usage; and barriers and perceptions, including a 10-point Likert scale rating of overall e-learning experience.

Ethical considerations

The study protocol was reviewed and approved by the institutional Ethics Committee of AIIMS Guwahati (M1/F3/2023 dated 20th February 2023). Participation was voluntary, and informed consent was obtained from all the participants before enrollment, and anonymity was assured.

Data collection procedure and statistical analysis

After obtaining institutional ethics approval, students were approached in classroom settings. The purpose of the study was explained, and written informed consent was obtained. The paper-based questionnaire was distributed and collected in the same session, with an average completion time of 10 to 15 minutes. No identifying information was recorded. Completed questionnaires were checked for completeness, and coded data was entered into the Microsoft Excel (Redmond, USA) spreadsheet and analyzed using IBM Corp. Released 2024. IBM SPSS Statistics for Windows, Version 30. Armonk, NY: IBM Corp. Descriptive statistics were used to summarize the demographic characteristics, device access, and e-learning usage patterns. The association between the categorical variables, such as gender, MBBS phase, and reasons for using e-learning or platform awareness, was tested using Pearson’s chi-square test or Fisher’s exact test. Binary logistic regression was performed to identify independent predictors of engagement in selected e-learning domains (theory, practical, and assessment), and results were expressed as odds ratios (ORs) with 95% confidence intervals. A p-value of less than 0.05 was considered statistically significant.

## Results

The study included 176 medical students (mean age 20.4 years), most of whom were male, 138 (78.4%), and in the first or second year, 79 (44.9%) and 65 (36.9%), respectively, with almost universal smartphone ownership in 161 (91.5%) and substantial prior computer training, 140 (79.5%). E-learning was widely used for theory (168, 95.5%), practical skills (145, 82.4%), clinical cases (141, 80.1%), assessments (94, 53.4%), and online tests (107, 60.8%). Online study materials were the main resource for theory, and YouTube was the most preferred resource for practical and surgical training (Table 1). Social media was commonly used for learning by 157 (89.3%). Awareness of institutional LMS was low (22, 12.5%), and only a few students attended free national courses such as NPTEL (12, 6.8%), while a majority attended paid online courses (107, 60.8%); however, about half of the participants used mobile applications for learning (Table 2). Reported barriers included the cost of gadgets and time constraints, while many students perceived that e-learning saved time, allowed convenient access, and helped keep up with the recent updates (Table 3). Gender was not associated with e-learning usage preference for theory, clinical cases, or mobile apps, but female students used e-learning for online tests and assessments significantly more than the males (p=0.02) (Table 4). Across MBBS phases, there were significant associations for clinical case learning, online tests, assessments, paid online courses, mobile app use, and STS awareness with higher values seen in senior years, whereas theoretical learning, social media use, and NPTEL attendance did not differ by academic phase (Table 5). Logistic regression tests were performed to identify independent predictors of e-learning engagement across theoretical and practical domains and for assessment. Assessment was found to be highly significant (c² = 25.337, p < 0.001). Three predictors that were found to have statistical significance for the assessment model were smartphone (OR=5.38, p=0.017), laptop (OR=2.85, p=0.005), and gender (OR=0.30, p=0.007). Marginal significance was found for theoretical learning by e-learning (c²=13.106, p=0.007), with smartphones found as significant predictors for using e-learning for theory (OR=23.81, p<0.001). No significant predictors were found for the practical model (c² = 6.261, p = 0.510). Gender, phase of MBBS, and prior computer training were not found to be significant predictors in our study. A Likert scale view on e-learning was obtained. A total of 97 (55.1%) had a score less than or equal to 5, while 79 (44.9%) had a score more than 5.

**Table 1:** Preferences of resource utilization for different domains of learning

Characteristics	n (%)
Resources utilized for theory	
Online study material	170(97.1)
Textbook	162(92)
E-books	148(84.1)
PowerPoint presentation	139(79)
Readymade study materials	128(72.7)
Class notes	118(67)
Online course module	101(57.4)
Resources utilized for practical	
You Tube videos	124(70.5)
Textbook	101(57.4)
E-books	67(38.1)
Online study materials	53(30.1)
Resources utilized for surgical techniques	
You Tube videos	98(55.7)
Textbook	52(29.5)
Online study materials	18(10.2)
E-books	8(4.5)

**Table 2:** Awareness on the use of different e-learning platforms by students

Platforms used	n (%)
Use of social media for learning	
Yes	158(90.3)
No	17(9.7)
The social media platform used for learning	
WhatsApp	6(3.4)
Telegram	59(33.7)
Facebook page	01(0.6)
LinkedIn	04(2.3)
YouTube	88(50.3)
Awareness about learning management system (LMS)	
Yes	22(12.5)
No	154(87.5)
Awareness about Short term studentship program (STS)	
Yes	136(77.3)
No	40(22.9)
Use of mobile applications for learning	
Yes	99(56.3)
No	77(43.8)
Attending courses like NPTEL	
Yes	12(6.8)
No	164(93.2)
Attend paid courses	
Yes	107(60.8)
No	69(39.2)

**Table 3:** Barriers and perceptions towards e-learning resources among medical undergraduates

Barriers Towards E-Learning	n(%)
Language barrier	05(2.9)
Poor knowledge of computer skills	14(8)
Cost of gadgets	28(15.9)
Cost of Internet	15(8.5)
Not able to utilize e-learning resources due to time constraint perceptions	24(13.6)
Saves time for understanding a topic	60(34.1)
Helps in keeping up with recent updates	31(17.6)
Freedom to access as per convenience	74(42)
Study materials are better than what is taught	11(6.3)

**Table 4:** Association between gender and reason for using e-learning platforms Table 4 summarizes the association between gender and reasons for using e-learning tools. No significant gender difference was found for the usage of e-learning for theory, clinical cases, and the use of mobile learning apps. However, female students use e-learning platforms for assessments more than males (c2 = 5.159, p-value = 0.02).

Reason for Using E-Learning	Male n (%)	Female n (%)	Total n (%)	c^2^(df)	p-value
For theoretical learning	131(94.9)	37(97.4)	145(82.4)	0.409(1)	0.52
For clinical cases	114(82.6)	27(71.1)	141(80.1)	2.498(1)	0.11
For online test/assessments	55(39.9)	23(60.5)	78(44.3)	5.159(1)	0.02
Uses mobile learning app	77(55.8)	22(57.9)	99(56.3)	0.053(1)	0.81

**Table 5:** Association between MBBS phases and e-learning use and awareness The association between MBBS phase year and various e-learning variables is summarized in Table 5. No significant association between the MBBS phases and the e-learning use was found for theoretical learning, use of social media, or for free online courses. However, significant associations were found for clinical case learning, online test assessments, paid online courses, mobile app usage, and awareness about STS.

E-Learning Variable	c^2^(df)	p-value	Interpretation
For theoretical learning	3.109(3)	0.37	Not significant
For clinical case learning	8.804(3)	0.03	Significant (­ usage in final years)
For online tests and assessments	15.245(3)	0.002	Significant (­ usage in final years)
Uses social media	4.425(6)	0.62	Not significant
Attends paid online courses	13.634	0.003	Significant (­ trend in final years)
Attends free online courses (like NPTEL)	1.224(3)	0.747	Not significant
Use of mobile applications for learning	10.582(3)	0.01	Significant. Second year students used mobile apps the most (71%)
Awareness about STS	17.298(3)	<0.001	­ trend in final years
Awareness about database	7.253(3)	0.06	Marginally significant

## Discussion

Our study investigated e-learning utilization and its predictors among Indian medical undergraduate students using the framework of self-directed learning and connectivism, which emphasizes learners' autonomy, motivation, and digital network connectivity.

Logistic regression analysis in our study identified device ownership, particularly smartphones and laptops, as significant predictors of engagement in the assessment domain, while smartphones alone significantly predicted theory-based engagement of students. The academic phase of MBBS and prior computer training were not significant predictors of overall engagement in digital learning behavior. This reflects the multifactorial nature of digital learning behavior in a rapidly evolving digital educational landscape in medical education [1,2]. The high ownership and accessibility of electronic devices as found in our study reflect the integration of digital tools into students’ academic routines, comparable to data from other Indian and international studies [3,4].

Globally, e-learning has become a core component of undergraduate medical education, with multiple studies demonstrating its acceptability and effectiveness for knowledge acquisition and learner satisfaction when blended appropriately with traditional teaching [1,2]. Studies such as “Brave New E-world” from the University of Michigan have shown that medical students preferentially use laptops, tablets, and commercial e-learning tools, especially for high-stakes exam preparations, and videos were the most popular modality and resource choice [3]. These findings align closely with our results, where students preferentially used online study materials and e-resources for theory and YouTube videos for practical skills and surgical techniques (Table 1). The strong preference for video content in our study population is consistent with the patterns described by the Generation Z learners who favor multimedia over text-based resources.

Within Asian countries, similar patterns with a notable increase in e-learning usage, facilitated by the mobile technologies, and with variations driven by the digital literacy and the infrastructural disparities have been observed [5,6]. Our findings correlate with these regional trends, particularly device access as a fundamental determinant of effective e-learning. The absence of significant predictors for practical domains aligns with global and regional studies, which point out that, even now, virtual platforms have a limited role in replicating hands-on clinical and procedural skill acquisition. This stresses the need for blended learning approaches. Like our study, other studies from India have also documented high awareness and use of e-learning resources among medical undergraduate students, but variability remains in the type and quality of resources accessed [5,7-9]. However, our study adds nuance by demonstrating device ownership and gender as predictors via logistic regression. This analytic approach extends beyond the descriptive studies, thus providing evidence for predictors of e-learning among Indian medical undergraduates.

Gender played a domain-specific role in our study. Among our study population, female students used e-learning platforms for online tests/assessments significantly more than males (Table 4). While no significant gender difference was found for the usage of e-learning for theory or clinical cases. This is consistent with the findings from global and Indian studies that female learners may preferentially use e-learning resources for assessments. This reflects the differing learning strategies, motivations, and domain-specific behavioral patterns among medical undergraduates [3,10].

The academic phase did not emerge as an independent predictor in our study (Table 5); however, there was a clear statistical difference in the type of resources used as the students advanced through the MBBS program. Senior MBBS students demonstrated higher usage of e-learning platforms for clinical case learning, assessments, and paid courses. Our findings are consistent with the findings from the global studies, which have described that in preclinical years, students rely on course-linked materials but later shift towards exam-oriented digital resources. The increased use of mobile applications by second-year students reflects the tendency of students to experiment with online learning. There was a lot of variation observed in the pattern of awareness of platforms and their utilization in our study. Low awareness about LMS and high awareness about student short-term scholarships (STS) were noteworthy. This aligns with efforts of students to nurture research competencies and autonomous learning.

In India, to encourage undergraduate medical students towards research and support e-learning, several initiatives have been taken. The national program on technology-enhanced learning (NPTEL) is a flagship government initiative to empower minds and foster online learning to achieve academic excellence. Several medical courses are available under NPTEL. The student short-term scholarship (STS) program of ICMR is an initiative to encourage undergraduate medical students towards research through part-time funded scholarships. In our study, only 6.8% of students attended free courses such as NPTEL, indicating minimal updates despite national-level promotion of these free online courses. This low engagement aligns with other studies, which documented barriers including lack of alignment within the medical curriculum, limited perceived relevance, and inadequate faculty endorsement (Table 2). In sharp contrast, about two-thirds of students (60.8%) reported attending paid online courses. This significant preference for specialized paid digital content aligns with global trends, such as the shift toward commercial resources (like UWorld or Pathoma) for preparing high-stakes examinations like USMLE Step 1. In India, platforms like Marrow and PrepLadder are popular among medical students due to their tailored content designed for the preparation of competitive exams [5].

Social media and YouTube were the most popular learning platforms in our study for theory, practical, and clinical cases. YouTube videos were the most preferred learning resource for learning practical and surgical techniques (70.5% and 55.7%, respectively, as shown in Tables 1, 2). This strong preference for videos is consistent with characteristics of Generation Z students, who tend to prefer multimedia learning resources over reading long texts. However, as noted in global literature, the quality of freely available medical videos can be highly variable, implying a need for faculty guidance to ensure reliable use in clinical training [4].

Perceptions and barriers of medical undergraduates in our study align with global and Indian literature [2,13,14]. Students in our study valued e-learning for convenience, time saving, and flexibility, and our findings are consistent with the SOAR analysis by Kumar et al. [7]. Around 44.9% rated e-learning > 5 on the Likert scale, yet many did not find the online resources better than what was taught in the classroom. This reinforces the findings from other studies that e-learning is best viewed as a complement, rather than a replacement, to traditional teaching methods, and students generally perceive a significant gap in the capability of e-learning to develop competencies across various domains [3,4,6,15,16].

Despite widespread acceptance and perceived benefits, the effective utilization of e-learning is hampered by technological, infrastructural, and psychological barriers, particularly in low- and middle-income countries (LMICs) [5,7-9,14,17]. While our study reported relatively low rates of difficulty arising due to the cost of internet or poor knowledge of computer skills, as our institute is a tertiary care center with free internet facilities for students, findings contrast sharply with challenges reported in other LMIC settings. Studies from LMICs have highlighted poor network connectivity as the most common barrier. In our study, time constraint was identified as a barrier to e-learning (Table 3). Our findings relate to other studies, where students have reported additional disadvantages of e-learning, such as eye strain, extended screen time, and distractions [5,13,14].

The strength of our study includes the use of multivariate logistic regression to identify independent predictors and analysis of resources and platforms across domains (theory, clinical, and assessment). Limitations of our study are data from single institutions, a cross-sectional design, possible self-report and recall bias, and a lack of direct linkage between e-learning patterns and academic performance. In addition, we did not assess the practices by different faculties, the quality of available e-content, or institutional policies, which may strongly influence utilization and impact.

## Conclusions

This study highlights that e-learning is highly accepted and widely utilized by undergraduate medical students in Northeast India, driven by near-universal ownership of smartphones and laptops. Engagement in digital learning, particularly for the assessment domain, is independently predicted by device ownership and gender. The utilization pattern of the resources is task and academic-year need-oriented. Senior students have a higher preference for paid specialized online courses. Students overwhelmingly prefer video lectures for learning complex topics and practical techniques, reflecting the preferences of Generation Z learners for multimedia content. The findings support self-directed learning behavior of students and emphasize e-learning as an essential adjunct to rather than a replacement for traditional teaching. This underscores the need to tailor gender- and phase-sensitive e-learning frameworks aligned with competency-based medical education goals.

## Figures and Tables

**Figure 1:**
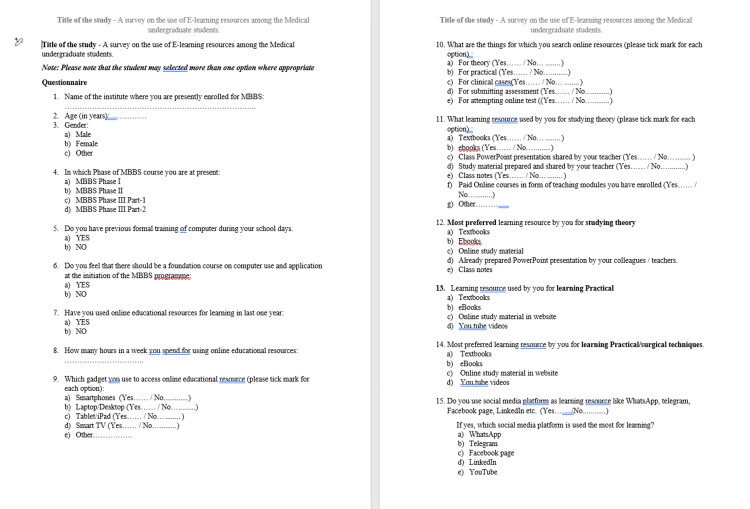
Questionnaire part 1

**Figure 2:**
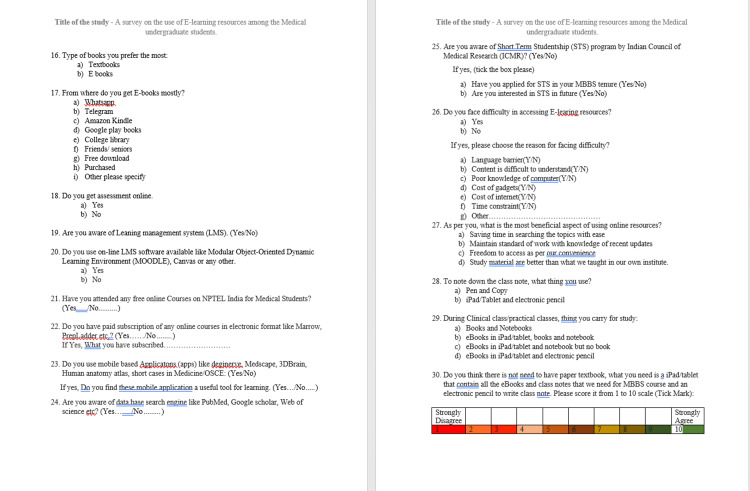
Questionnaire part 2
